# A Radiation Oncology Department Open House Improves Medical Students’ Reported Familiarity With and Interest in the Specialty

**DOI:** 10.7759/cureus.108856

**Published:** 2026-05-14

**Authors:** Brady S Laughlin, Sean T McCauley, Jonathan B Ashman, Randa Tao, Joshua R Niska, Lisa A McGee

**Affiliations:** 1 Radiation Oncology, Mayo Clinic Arizona, Phoenix, USA

**Keywords:** medical education, medical student engagement, open house, radiation oncology education, radiation oncology workforce

## Abstract

Background

Radiation oncology (RO) plays a critical role in the multidisciplinary care of cancer patients. As an integral component of comprehensive oncologic care, medical students should have meaningful exposure to the field to develop a foundational understanding of radiation medicine concepts. Because physicians across nearly all specialties encounter patients with cancer, a basic understanding of radiation therapy is essential for delivering high-quality, patient-centered care.

Methods

A single-institution open house was conducted for local medical students with the goal of increasing awareness and knowledge of RO. The event included lectures describing an overview of RO and residency training, as well as technical considerations such as common RO indications and radiation treatment planning and delivery modalities. This was followed by a department tour to see and learn about RO treatment machines, including linear accelerators, proton therapy gantries, a CT simulator, and a brachytherapy afterloader device, as well as an interactive question-and-answer session. Participants were invited to complete an anonymous post-event survey consisting of six pre-/post-comparison questions (Likert scale: 1 = Not at all familiar to 5 = Very familiar), five questions regarding the impact of the open house, and general feedback. Survey items evaluated baseline exposure to RO, familiarity with the role of the radiation oncologist and aspects of the field, comfort asking questions, and interest in further exposure.

Results

Fifteen students attended the open house, and 12 completed the survey: nine (75%) MS1, one (8.3%) MS2, and two (16.7%) MS3. Baseline familiarity with RO was low (mean 2.5), with seven (58.3%) reporting no or minimal familiarity and six (50%) indicating no prior exposure. Following the open house, understanding of the role of a radiation oncologist improved (mean 2.3-4.1), as did knowledge of clinical indications (2.1-3.5), treatment planning (2.0-3.9), the multidisciplinary nature of care (2.8-3.8), and radiation therapy delivery modalities (1.8-3.8). Participants reported increased understanding of the specialty (mean 4.7), awareness of technology (4.6), comfort asking questions (4.6), and interest in RO (4.3). All respondents expressed motivation to pursue additional exposure, with the highest interest in clinical shadowing (100%), research opportunities (91.6%), and mentorship or career advising (91.6%). Favorable highlights of the event were the departmental tour and question-and-answer session.

Conclusions

A structured open house notably improved medical students’ understanding, awareness, and interest in RO. This event represents a feasible, scalable approach to early medical student engagement. This approach can be reproduced in other RO departments to enhance medical student engagement, knowledge, and continued engagement with the specialty.

## Introduction

Gaps exist in medical school curricula with regard to the principles of multidisciplinary oncology management [1]. The Liaison Committee on Medical Education (LCME), the national accrediting body for medical schools in the United States, defines basic curriculum content standards for medical student education. LCME standards state that medical school curricula must cover fundamental concepts, which include organ systems, the life cycle, prevention, symptoms, signs, differential diagnosis, and treatment planning, as well as the scientific method and the approach to translational research. Beyond providing this framework, the LCME does not make recommendations for specific topic content, such as oncology, to be taught during medical school [2].

Likewise, the United States Medical Licensing Examination (USMLE) provides a vague Content Outline for candidates’ exam preparation. This Content Outline provides a list of study topics by organ systems. Oncology is not included as a stand-alone topic but is woven through each organ system by listing the common neoplasms that can be encountered for each organ system. The Content Outline does not specify mastery of treatment approaches to these neoplasms [3].

Since medical school curriculum accreditation requirements and the USMLE Content Outline omit the main concepts of oncologic care, this creates a potential knowledge gap in medical education, where medical students may not be exposed to multidisciplinary oncologic care, which includes medical oncology, surgical oncology, and radiation oncology (RO). Students may be exposed to systemic treatment options, such as chemotherapy medications, through pharmacology and basic oncology lectures and to surgical excision of tumors through required surgical clinical rotations. However, RO exposure and teaching during medical school are largely lacking unless the individual medical school intentionally includes this as an additional topic to round out medical student education [4].

Currently, only approximately 40% of medical schools are affiliated with an RO department that also has an RO residency program. Even fewer schools (4%) have RO-specific lectures in the first two preclinical years, even though most curricula include general oncology concepts [4].

Because approximately 50-60% of the two million cancer patients diagnosed annually in the United States will require radiotherapy, most physicians or physicians-in-training will encounter patients who are either currently undergoing radiotherapy or who have received it in the past [5,6]. A basic understanding of RO and its impact on patients is therefore essential for physicians to provide holistic care. Interventions to increase medical students’ knowledge and awareness of RO are therefore critical. The literature demonstrates that when medical students are provided focused teaching on RO principles, there is increased understanding of multidisciplinary cancer care and appropriate referrals [7]. We highlight an initiative of a single-institution open house introducing the specialty to medical students and providing them with education on basic RO principles.

## Materials and methods

Study design

This study evaluated the impact of an RO open house developed at a single institution to increase awareness and understanding of the specialty among local medical students. The initiative was designed as an educational intervention followed by an anonymous post-event survey incorporating paired pre- and post-event self-assessment items.

The primary objective of this study was to evaluate changes in self-reported familiarity with RO domains before and after an open house. Secondary objectives included assessing participant interest in further RO engagement and identifying qualitative strengths and weaknesses of the event format.

Study setting and participants

Medical students from five local medical schools were invited to attend the RO open house through institutional group communication channels. An open house flyer was emailed to medical school deans, medical school administrators, and student interest group leaders for wider distribution to all students at each local medical school. Participation was voluntary, and no incentives were provided for attendance or survey completion. The event was open to students regardless of prior exposure to medical oncology or RO.

Intervention

The open house was a three-hour event structured to provide students with a broad introduction to the field of RO and the residency training pathway. Six faculty members, including the residency program director, the associate program director, two medical student clerkship co-directors, a physician faculty member at large, and a physics faculty member at large, participated in the open house, as well as all four advanced RO medical residents, who represent all resident physicians in the RO residency training program. The event started with a 30-minute introduction session, where faculty and resident physicians introduced themselves and explained why they chose to pursue RO specialty training after medical school. Following faculty and resident introductions, the residency program director gave a 15-minute overview lecture of RO residency training, and the two medical student clerkship co-directors led a 15-minute lecture overview of radiation treatment, common clinical indications, treatment planning and delivery, and the multidisciplinary role of RO in cancer care. Participants then completed a one-hour department tour to see RO treatment machines, including linear accelerators, proton therapy gantries, a CT simulator, and a brachytherapy afterload device. The tour was narrated by a medical physicist, with input from the faculty and resident physicians, to explain how each of the machines worked and how they could be used in cancer care. The event concluded with a one-hour interactive question-and-answer session with faculty and residents.

Survey instrument

Following the open house, participants were invited to complete an anonymous survey assessing their understanding of multiple domains within RO using paired pre- and post-event Likert scale items (1 = not at all familiar to 5 = very familiar). Topics assessed included general knowledge of RO, common clinical indications, treatment planning and delivery, multidisciplinary collaboration, and radiation therapy modalities.

Additional survey questions evaluated baseline exposure to the specialty, familiarity with the role of a radiation oncologist and other members of the care team, and perceptions of the open house experience. Likert scale questions also assessed the perceived impact of the event on understanding of the field, comfort in asking questions, and interest in future exposure to RO. Higher scores indicated greater familiarity, agreement, or interest. Future engagement opportunities were assessed using multiple-selection questions. Open-ended questions solicited feedback regarding the most helpful aspects of the event, opportunities for improvement, and additional comments.

The survey tool was investigator-developed; a validated survey instrument assessing RO knowledge from a departmental open house was not available to collect information specific to this initiative.

Statistical analysis

Descriptive statistics were used to summarize survey responses. Paired pre- and post-event Likert scale responses were compared descriptively to assess changes in self-reported understanding of RO concepts. Free-text responses were reviewed qualitatively to identify common themes related to the strengths of the open house and opportunities for future improvement.

## Results

Fifteen medical students from 4/5 of the local medical schools attended the RO open house. The RO open house survey instrument provided to the event attendees is included in Table 1. Twelve of 15 participants (80%) completed the post-event survey. Baseline familiarity with RO was low, with a mean score of 2.5 on a 5-point Likert scale. Seven (58.3%) reported no or minimal familiarity, and six (50%) indicated no prior exposure. Following completion of the open house, participants indicated a higher understanding of multiple areas (Table 2). Participants reported a stronger understanding of the role of a radiation oncologist, with an increased mean score from 2.3 to 4.1. Knowledge of clinical indications also increased, with a mean score increasing from 2.1 to 3.5. Additionally, there were improvements across other domains, including treatment planning (mean 2.0-3.9), the nature of multidisciplinary care (mean 2.8-3.8), and radiation therapy delivery modalities (mean 1.8-3.8).

**Table 1:** Open house survey tool MS, medical student

Survey item	Response scale
What is your current level of training?	MS1-MS4; Other (categorical)
Prior to attending the open house, how familiar were you with radiation oncology?	1-5 Likert (1 = Not at all familiar, 5 = Extremely familiar)
Have you had any prior exposure to radiation oncology?	Multiple choice (e.g., None; Preclinical didactics; Clinical elective; Research; Other)
Please rate your understanding of the following topics BEFORE the open house - Role of radiation oncology in cancer care	1-5 Likert (1 = Not at all familiar, 5 = Extremely familiar)
Please rate your understanding of the following topics AFTER the open house - Role of radiation oncology in cancer care	1-5 Likert (1 = Not at all familiar, 5 = Extremely familiar)
Please rate your understanding of the following topics BEFORE the open house - Common clinical indications	1-5 Likert (1 = Not at all familiar, 5 = Extremely familiar)
Please rate your understanding of the following topics AFTER the open house - Common clinical indications	1-5 Likert (1 = Not at all familiar, 5 = Extremely familiar)
Please rate your understanding of the following topics BEFORE the open house - Basics of treatment planning and delivery	1-5 Likert (1 = Not at all familiar, 5 = Extremely familiar)
Please rate your understanding of the following topics AFTER the open house - Basics of treatment planning and delivery	1-5 Likert (1 = Not at all familiar, 5 = Extremely familiar)
Please rate your understanding of the following topics BEFORE the open house - Multidisciplinary collaboration	1-5 Likert (1 = Not at all familiar, 5 = Extremely familiar)
Please rate your understanding of the following topics AFTER the open house - Multidisciplinary collaboration	1-5 Likert (1 = Not at all familiar, 5 = Extremely familiar)
Please rate your understanding of the following topics BEFORE the open house - Types of radiation therapy (e.g., photons, protons, brachytherapy)	1-5 Likert (1 = Not at all familiar, 5 = Extremely familiar)
Please rate your understanding of the following topics AFTER the open house - Types of radiation therapy (e.g., photons, protons, brachytherapy)	1-5 Likert (1 = Not at all familiar, 5 = Extremely familiar)
The open house increased my understanding of what radiation oncologists do.	1-5 Likert (1 = Strongly disagree, 5 = Strongly agree)
The open house improved my awareness of the technology used in radiation therapy.	1-5 Likert (1 = Strongly disagree, 5 = Strongly agree)
The open house helped me better understand the patient experience in radiation oncology.	1-5 Likert (1 = Strongly disagree, 5 = Strongly agree)
I felt comfortable asking questions and interacting during the open house.	1-5 Likert (1 = Strongly disagree, 5 = Strongly agree)
The information was presented in an engaging and understandable way.	1-5 Likert (1 = Strongly disagree, 5 = Strongly agree)
How did the open house affect your interest in radiation oncology as a specialty?	1-5 Likert (1 = Much less interested, 5 = Much more interested)
After attending the open house, how likely are you to pursue additional exposure to radiation oncology?	1-5 Likert (1 = Very unlikely, 5 = Very likely)
Which types of follow-up opportunities would you be interested in?	Free text
What aspects of the open house were most helpful for your learning?	Free text
What aspects could be improved for future open houses?	Free text
Additional comments or suggestions?	Free text
What is the optimal time of year for an open house for MSs?	Free text

**Table 2:** Change in self-reported understanding before and after the radiation oncology open house

Domain	Pre-open house mean	Post-open house mean	Mean change
Role of the radiation oncologist	2.3	4.1	1.8
Common clinical indications for radiation therapy	2.1	3.5	1.4
Treatment planning and delivery	2	3.9	1.9
Multidisciplinary collaboration	2.8	3.8	1
Radiation therapy modalities (e.g., photons, protons, or brachytherapy)	1.8	3.8	2

The open house was positively received, with respondents reporting a mean score of 4.6 for comfort asking questions and a mean score of 4.7 for interest in the specialty. All respondents reported interest in learning more about the specialty. Regarding future opportunities, all respondents (100%) reported interest in additional exposure, with the highest eagerness for clinical shadowing, followed by research opportunities and mentorship and career advising.

Qualitative feedback supported the positive impact of the open house. When asked about aspects of the open house that were most helpful, six comments were provided, with the tour and Q&A receiving the highest praise. One comment noted that it was helpful to learn the attending and resident physician perspectives on why RO was pursued as a specialty. Three respondents provided comments expressing gratitude and acknowledgment of the helpfulness of the event.

Opportunities for improvement were also identified. When asked about aspects of the event that could be improved, two students provided feedback that the day-to-day life of a radiation oncologist was not addressed in a clear and consistent manner. An additional comment suggested having breakout sessions for the question-and-answer session.

In addition to survey data, the utility of the open house can be assessed through continued medical student interest. Of the 15 open house attendees, four students contacted the program afterward to set up educational opportunities to further enhance RO learning and exposure. Two students scheduled preclinical observerships, and two additional students scheduled both preclinical observerships and research rotations.

## Discussion

The purpose of an RO open house is to provide medical students with immersion in a lesser-known specialty and increase awareness and understanding of the role a radiation oncologist plays in the multidisciplinary care of cancer patients. A basic understanding of RO at the medical student level is essential, as most will go on to care for cancer patients who have received radiotherapy. Accurate knowledge of the specialty is important to avoid inaccurate perceptions or biases that may result from limited, indirect exposure. Additionally, exposure to RO may be a first step toward medical students pursuing the specialty as a career and, therefore, may be useful for RO residency recruitment. Based on our program’s initial success in both virtual and in-person open houses in medical student exposure, we sought to recreate the in-person open house event and survey the medical students to better understand the utility of this event from the medical student perspective, as well as identify areas of improvement to further enhance the medical student RO open house experience [8]. These survey data, therefore, represent the medical student perspective of our departmental RO in-person open house.

The survey reported here demonstrated that the RO open house was effective in improving self-assessed medical student knowledge of the specialty, including better understanding of the role of a radiation oncologist, common clinical indications for radiation therapy, radiation treatment planning and delivery, radiation modalities, and multidisciplinary collaboration. To date, there is no published literature assessing the utility of a medical student open house from the perspective of the medical student, which makes this study unique. The major advantage of medical student outreach through a departmental open house is the small-scale approach that makes this a feasible activity at the level of the individual department. It is a straightforward, single-day event that can be conducted with limited resources. While the literature does not provide specific evidence regarding the utility of open house events, there are a variety of other medical student engagement initiatives reported to also result in increased interest and awareness of the specialty. Tsui et al. conducted a radiation oncologist-driven tumor board shadowing experience at three medical schools. Participants felt that the tumor board shadowing provided better or similar educational content, specialty exposure, and RO experience compared with clinical rotations. Nearly half of the participants learned that there was more multidisciplinary collaboration in cancer patient care than what was previously realized [7]. Similarly, virtual and in-person education programs designed for medical students showed improved RO knowledge through case-based discussions and other RO topics [9-12]. Additionally, an immersive six-week RO summer studentship was rated as a high-yield educational activity that led to increased interest in the field of RO [13]. Mentorship in RO has also demonstrated utility in maintaining and increasing interest in RO as a career [8,14,15]. The data from our in-person open house experience serves as an additional way for an RO department to conduct medical student engagement and education, in addition to these other potential options.

While each reported medical student engagement initiative has demonstrated value in RO education early in the physician’s career, efforts to include RO education in medical student education are still currently conducted at the local level. To date, RO has not found its place in standard medical school curricula, nor is it a required topic for licensing board examinations [2,3]. Therefore, individual program efforts to enhance medical student education and engagement within the specialty should be pursued. This study on an RO departmental open house represents one feasible, scalable approach for an individual program to adopt.

Our medical student in-person open house experience also highlights the potential utility of early specialty exposure in creating medical student interest in RO and possible future residency recruitment. Our initial report of a comprehensive approach to medical student engagement showed early potential success of an in-person departmental open house [16]. In the initial experience, four out of 10 open house attendees sought additional RO clinical exposure, leading to one student requesting an RO lecture to a medical student interest group the following academic year, two students pursuing preclinical observerships and research rotations, one of whom was awarded the ASTRO Medical Student Fellowship Award, and another student who pursued a fourth-year audition rotation and applied to our residency program the following academic year [16]. Likewise, the experience from this current open house has generated medical student interest in additional clinical experience. Of the 15 attendees, four students are continuing to pursue RO education through preclinical observerships, two of whom have also scheduled research rotations. Considering this positive impact two years in a row, our RO residency program plans on continuing the in-person open house efforts to further enhance medical student exposure to the specialty and potentially enhance residency recruitment.

This study has several limitations. As a single-institution experience with voluntary participation, the findings may be subject to selection bias and may not be generalizable to other institutions or medical student populations. Participants who chose to attend the open house may have already had a greater baseline interest in oncology or RO, which could influence perceptions of the event and post-survey responses. Another limitation is that there was no control group for comparison. Additionally, the sample size was limited, which results in statistics that are underpowered to show significance. Hence, this study is limited by descriptive analyses, which can summarize data but have limitations in drawing conclusions about relationships, causality, and generalizability. Outcomes data were based on self-reported familiarity and perceptions rather than objective assessments of knowledge acquisition or long-term career impact. Attendance at the open house was largely MS1 students (75%); therefore, it is difficult to determine how much of the educational impact was simply due to the delivery of basic information to novices. The use of retrospective pre- and post-event Likert scale evaluations also introduces response bias, as it has been shown to inflate gains. Despite these limitations, the study provides valuable insight into the potential role of structured early exposure initiatives in improving medical students’ understanding of RO and supporting future engagement with the specialty.

## Conclusions

A structured open house substantially improved medical students’ knowledge, understanding, awareness, and interest in RO. The event provided early, meaningful exposure to key concepts and the clinical role of the specialty within cancer care. This type of initiative represents an effective strategy for fostering medical student engagement and informing future career consideration.
